# Clinical Evidence of Retrograde Axonal Growth in Chronic Brachial Plexus Injury

**DOI:** 10.7759/cureus.62424

**Published:** 2024-06-15

**Authors:** Mohammed Tahir, Leonardo D Lanzarin, Bertelli A J

**Affiliations:** 1 Orthopaedic Surgery, Hospital Governador Celso Ramos, Florianopolis, BRA; 2 Trauma and Orthopaedics/Hand Surgery, Hospital Governador Celso Ramos, Florianopolis, BRA; 3 Surgery, Universidade Federal de Santa Catarina, Florianopolis, BRA

**Keywords:** nerve regeneration, axonal regeneration, brachial plexus injury, nerve injury, retrograde neural regeneration

## Abstract

Nerve axons grow from proximal to distal after axonometric injury; however, they have been seen to regenerate via alternate routes, with some also demonstrating retrograde growth in neuromas. We present the case of a 33-year-old male with a 16-year-old traumatic brachial plexus injury presenting with neuropathic pain and isolated spontaneous recovery. Following a successful pre-operative anaesthetic block, a neurectomy of the median and ulnar nerves was planned for pain relief. Intraoperatively, median nerve stimulation resulted in muscle contractions in the pectoralis major (PM) and extensor carpi radialis brevis (ECRB). This was confirmed by electrical and mechanical stimuli. Histological analysis confirmed the presence of viable axons in the median nerve despite no distal nerve function. Post-surgery motor activity was preserved. A plausible explanation for the intraoperative observations, suggesting neural connectivity between the median nerve and PM and ECRB, would be retrograde growth into various nerve pathways. Alternative explanations such as axonal bifurcation, light anaesthesia, or anatomical variations were considered but the evidence favoured retrograde axonal regrowth. These findings challenge conventional understanding and offer potential new approaches to nerve reconstruction.

## Introduction

Typically, after nerve division and repair, axons cross the suture line from proximal to distal in an antegrade way [1,2]. Leis et al. first reported eliciting motor potentials in the first dorsal interosseous (DIO1) by electrical stimulation of the proximal end of an excised ulnar nerve neuroma at the elbow, despite a 2cm gap [3]. They explained this by a possible retrograde pathway for regenerating axons that grew via undetected anomalous branches or fibrous adhesions. Bertelli et al. observed contractions in pectoralis major (PM) from distal median nerve stimulation in a complete plexus lesion and also suggested an alternate retrograde regrowth of axons [4]. Histology confirmed viable axons in the median nerve despite absent hand function.

In the case we present, sequential surgical crush stimuli to the median nerve not only produced contractions of PM but also in extensor carpi radialis brevis (ECRB). Hence, muscle activation occurred not only proximal to the crush stimulus, but also distally. This represents new and thought-provoking findings.

## Case presentation

Our patient, a 33-year-old male presented with neuropathic pain following a right-sided, 16-year-old traumatic C5-T1 brachial plexus injury (TBPI). This was thought to be a mixture of pre and postganglionic. We did not treat him at primary presentation. He exhibited spontaneous recovery of biceps, PM, and ECRB (antigravity power in all), as well as elbow flexion. There was no median or ulnar nerve function. An anesthetic block in the median and ulnar nerves (4 ml of 0.5% Neocaine) led to pain relief in the hand, prompting us to proceed with a neurectomy of both in an attempt to establish pain relief. Our ideal choice of intervention would have been a dorsal root entry zone rhizotomy (DREZotomy), but this was not available to the patient due to funding constraints.

**Figure 1:**
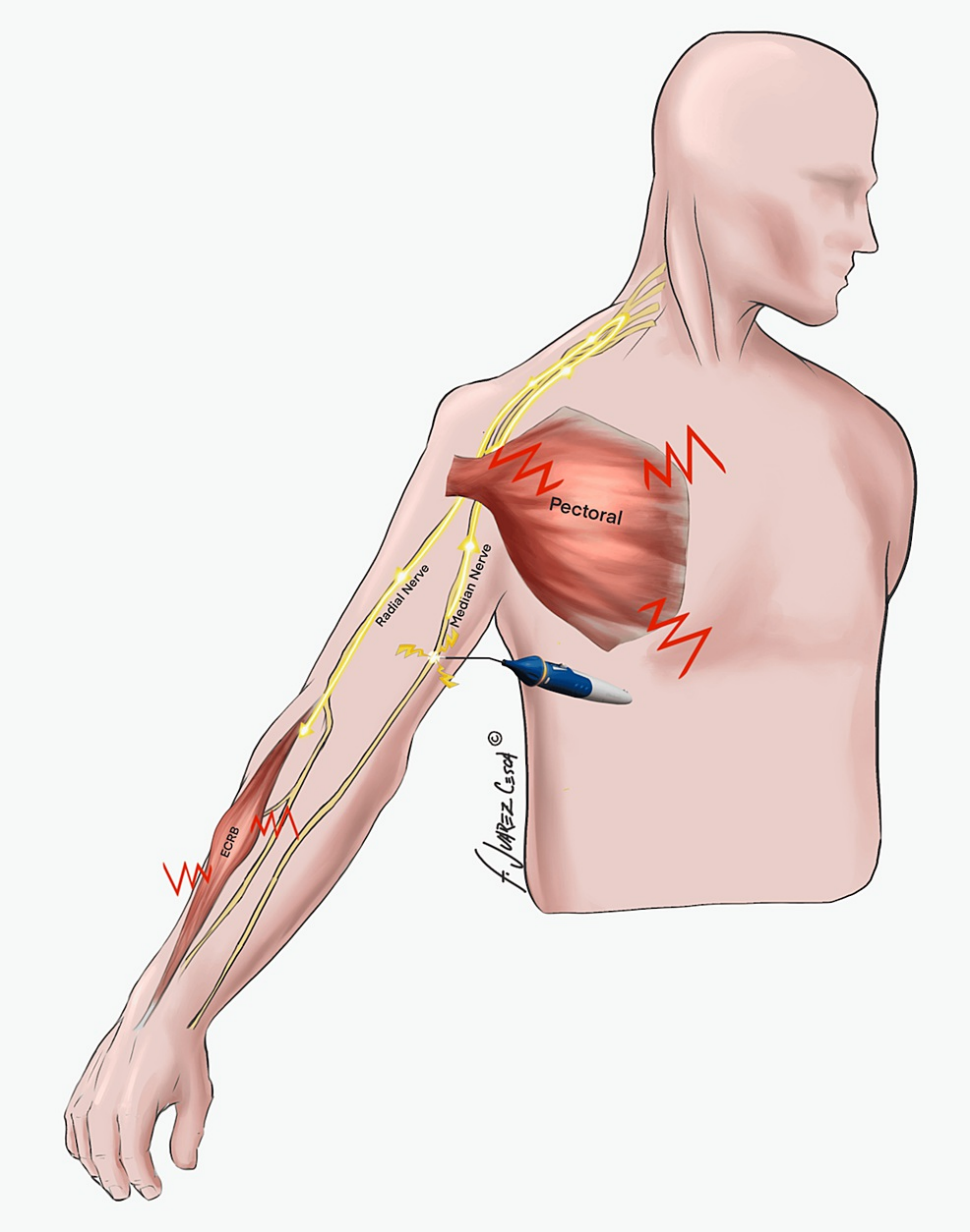
Schematic representation of the intraoperative clinical findings with nerve stimulation. Image Credit: Dr Bertelli et al. All rights reserved.

Under general anaesthesia without muscle paralysis, we approached the medial brachial bundle via a 10 cm longitudinal incision. The median nerve was dissected, isolated with vascular loops and stimulated with an insulated needle connected to a nerve locator (B. Braun, Melsungen, Germany). Clear contractions of PM and ECRB were observed, as represented schematically in Figure 1. To further eliminate the possibility of inadvertent electrical stimulation, we mechanically stimulated the median nerve by crush injury. Again, we observed clear twitches in PM and ECRB. Further distal crush stimuli produced no contractions, whereas more proximal crush stimuli elicited further muscle contractions as shown in Video 1.

**Video 1:** Concomitant muscle contraction in pectorals major and extensor carpi radialis brevis with median nerve stimulation

This was repeated in the ulnar nerve where no muscle contractions were observed. A neurectomy was performed in both, followed by resection of an 8 cm long segment of the median and ulnar nerve were excised and fixed in 10% formalin for histological analysis. The proximal stumps were buried into local muscle tissue.

**Figure 2:**
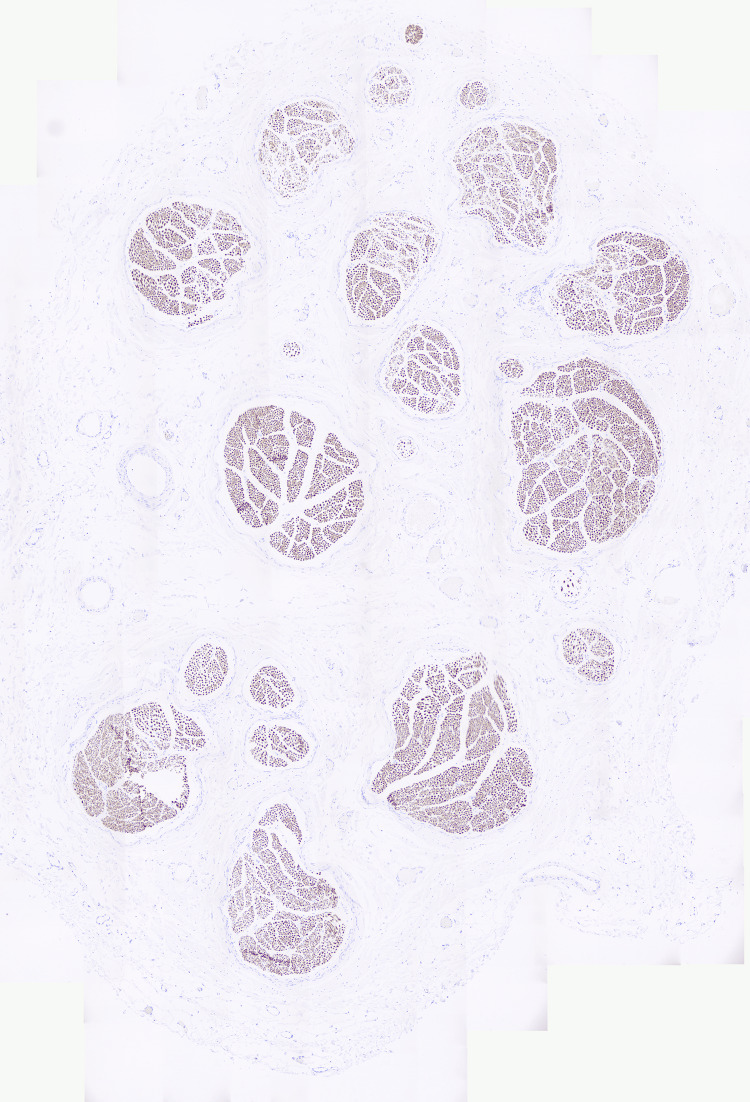
Histological immunohistochemistry for neurofilament demonstrated viable axons in the median nerve (magnified 200×).

One week postoperatively, our patient reported a 30% reduction in constant pain and 70% in shooting pain. Motor activity in the biceps, PM, and ECRB were maintained with no loss of power. Subsequent histological studies demonstrated the presence of neurofilament in the median nerve, indicating the presence of viable axons, though of course, this does not indicate the direction of these functioning axons, merely the presence (Figure 2).

## Discussion

Firstly, it is imperative to stress that the nature of this article is not to discuss our surgical strategy but rather to report on the intraoperative findings. We hypothesize that viable regenerating axons in the median nerve looped back at an unknown point along their course and subsequently into PM and ECRB. This would require retrograde axonal growth into the medial contribution of the median nerve, medial cord, and finally into the medial pectoral nerve. In the case of ERCB, this would require retrograde growth via either medial or lateral contribution to the median nerve, any of the trunks, and finally via any of their posterior divisions into the radial nerve. The pertinent feature that reinforces the idea of axonal retrograde loops is that more proximal but not distal crush stimuli would further elicit responses, as the latter would not elicit a novel response in retrograde axons, but proximal stimuli would. Postoperatively, preserved motor function in PM and ECRB implied the main innervation stemmed directly through neuromas in continuity. 

Alternative explanations for retrograde axonal regrowth include: (a) bifurcation of axons into the medial pectoral or radial nerve and the median nerve at the level of the primary injury site, (b) light anaesthesia, or (c) anatomical variants. 

Axonal bifurcation and misdirection of axons at the primary injury site of the median nerve axons might produce a stimulus in a sibling axon ending in PM or ECRB. However, we are unaware of any previous reports demonstrating co-stimulation of collateral axons, and it is not a typical finding in chronic plexus lesions in obstetric cases or adults. In the case of a light anesthetic, painful stimuli can cause a motor response. However, we did not notice other neural responses, namely in biceps or ulnar nerve crushing. We also confirmed the absence of hemodynamic changes. As for the latter, ECRB activation following median nerve stimulation could be explained by anatomical variations with possible connections between the median and radial nerve in the arm. However, here we would expect further distal crush stimuli to also produce ECRB activation, and this was not observed. 

In an electromyography (EMG)-based case series by Roth and Egloff-Baer in 10 patients with nerve lesions, latency responses were observed in a manner that could only be explained by the presence of motor axon loops, which they suggested likely due to congenital anomalies [5]. Neural anastomosis between the median and medial pectoral nerves, however, has not been described in the literature [6]. Furthermore, unlike the ulnar nerve, radial-median anastomoses have also not been reported.

Retrograde axonal growth is not a novel concept, first being cited by Cajal’s work in 1928 [1]. Using electron microscopic studies, Friede and Bischhausen demonstrated retrograde growth begins as soon as the first regenerative sprouts are formed [7]. Scadding and Thomas further demonstrated this with histological analysis of neuromas in resected rat sural nerves, proposing these retrograde pathways might play a role in motor activity, mechanosensitivity, and adrenaline sensitivity [8]. Of course, a key difference in our findings is that ours occur in a continuous nerve lesion. 

We know peripheral nerve regeneration is commonly misguided [9]. It is plausible that those directed backwards grow along tubules of adjacent axons that have undergone Wallerian degeneration, where macrophage-mediated degradation of the basal membrane occurs [10]. The elimination of these physical constraints inside the nerve could allow for retrograde elongation. 

Furthermore, two recent experimental rat studies demonstrated retrograde axons regenerating to reinnervate adjacent muscles. Bertelli et al. successfully performed ulnar nerve to posterior interosseous nerve (PIN) transfers via the superficial radial nerve (SRN), evidenced by both immunostaining and electrical stimulation post recovery [11]. A similar experiment in the hindlimb by Alzahrani et al. also demonstrated this in an obturator to saphenous nerve transfer, with muscle reinnervation via the femoral nerve [12]. This was also evidenced by immunostaining and optical tissue-clearing methods.

## Conclusions

The phenomenon reported here escapes traditional thinking of nerve regeneration and axonal elongation. We suggest retrograde axonal regrowth as a plausible explanation, where retrograde axon sprouts elongate via endoneural tubes with disrupted basal membranes. The potential clinical application of our findings relates to the reconstruction of nerve lesions. For instance, in a radial nerve lesion, the superficial radial nerve may be looped backward and connected to the proximal stump of the radial nerve, where axons would grow anterogradely and then retrogradely to reach the posterior interosseous nerve, thus avoiding nerve grafting. As with any new alternative for repair, the quality of recovery needs to be tested experimentally.
